# Network Analysis of Competitive State Anxiety

**DOI:** 10.3389/fpsyg.2020.586976

**Published:** 2021-01-11

**Authors:** Richard Mullen, Eleri Sian Jones

**Affiliations:** ^1^Division of Sport, Health and Exercise Sciences, Brunel University London, London, United Kingdom; ^2^School of Sport, Health and Exercise Science, Bangor University, Bangor, United Kingdom

**Keywords:** anxiety, network analysis, predictability, community detection, graph theory, state anxiety

## Abstract

Competitive state anxiety is an integral feature of sports performance but despite its pervasiveness, there is still much debate concerning the measurement of the construct. Adopting a network approach that conceptualizes symptoms of a construct as paired associations, we proposed re-examining competitive state anxiety as a system of interacting components in a dataset of 485 competitive athletes from the United Kingdom. Following a process of data reduction, we estimated a network structure for 15 items from the modified Three Factor Anxiety Inventory using the graphical LASSO algorithm. We then examined network connectivity using node predictability. Exploratory graph analysis was used to detect communities in the network and bridge expected influence calculated to estimate the influence of items from one community to items in other communities. The resultant network produced a range of node predictability values. Community detection analysis derived three communities that corresponded with previous research and several nodes were identified that bridged these communities. We conclude that network analysis is a useful tool to explore the competitive state anxiety response and we discuss how the results of our analysis might inform the assessment of the construct and how this assessment might inform interventions.

## Introduction

The measurement of competitive state anxiety (CSA) has been the subject of much debate in the sport psychology literature (Hardy, 1997; Mellalieu et al., 2006). While long acknowledged as a multidimensional construct (Martens et al., 1990; Cox et al., 2003), there have been important strides made toward understanding the exact nature of that multidimensionality to better understand the function of the construct. For example, Cheng et al. (2009) presented a model comprised of cognitive and physiological anxiety and a regulatory dimension, included to reflect the adaptive nature of the competitive anxiety response. A unique feature of Cheng et al.’s model is the differentiated structure of cognitive and physiological anxiety, designed to account for the unique processes subsumed within these dimensions. Specifically, the full model includes three higher order dimensions reflected by five lower order subcomponents; cognitive anxiety, reflected by worry and self-focused attention; physiological anxiety, reflected by autonomic hyperactivity and somatic tension and the regulatory dimension consisting of a single subcomponent, perceived control. To measure their model Cheng et al. developed the Three Factor Anxiety Inventory (TFAI). Initial testing failed to support the predicted hierarchical structure and Cheng et al. settled on a three-factor fit comprising cognitive anxiety, physiological anxiety and perceived control. Further support for the predictive validity of the model was established in subsequent research (Cheng et al., 2011; Cheng and Hardy, 2016). In both studies, the regulatory dimension played a key role in the dynamics of the anxiety response.

Jones et al. (2019) extended the work of Cheng and associates by respecifying the structure of the CSA model. From a conceptual standpoint, Jones et al. (2019) suggested that the self-focus subcomponent of the cognitive anxiety dimension proposed by Cheng et al. failed to recognize the commonly accepted multidimensional nature of this construct, which is more typically composed of public and private facets (Fenigstein et al., 1975; Geukes et al., 2013). In addition to specifying a structure that fully differentiated private and public self-focus, Jones et al. (2019) used a novel approach to model specification and measurement. Rather than adopt the reflective approach of classic test theory, where variation in scores on measures is a function of the true score and error, Jones et al. (2019) adopted a hybrid approach, consisting of reflective and formative measurement. In formative models, variables are viewed as composites of indicators, a notion Jones et al. (2019) applied to a higher-order factor structure in which the first order latent constructs of worry, private self-focus, public self-focus, somatic tension, autonomic hyperactivity and perceived control, were measured by reflective indicators. Each of these constructs had a unique theme common to all the items measuring it (Diamantopoulos and Winklhofer, 2001). The first order constructs served as formative indicators for the second-order latent variables, the cognitive, physiological and regulatory dimensions. Jones et al. (2019) specified these models as formative “as the direction of causality flows from the first to the second order constructs” (Jones et al., 2019, p. 43). In a series of studies, Jones et al. (2019) provided initial support for a 25-item representation of their model.

The work of Cheng and Jones and respective associates has significantly advanced the measurement of CSA. Despite these advances, the status of both reflective and formative measurement models is the source of much discussion, with most of the debate focused on the reasons for favoring one or other approach (Schmittmann et al., 2013). Amid this debate, others have sought alternative means of modeling psychological responses. Network analysis has emerged as an alternative to more traditional approaches to model development and measurement and sport psychologists could benefit from a consideration of the network structure of the phenomena they seek to understand. The network perspective views mental states as a complex system of interacting symptoms (Borsboom, 2017). From this perspective, the causal interplay between symptoms constitutes the mental construct (Fried et al., 2017). This view stands in contrast to the more common approach in which the construct is considered to be the latent cause of the thoughts and feelings that reflect its presence. From the network standpoint, CSA can be viewed as the emergent consequence of the interactions among its constituent elements (Schmittmann et al., 2013) and latent constructs are not necessary to explain how the items in a questionnaire covary. These interactions are depicted in a network and studying the construct means studying the architecture of the network. As Schmittmann et al. note, “the relation between observables and the construct should not be interpreted as one of measurement, but as one of mereology: the observables do not measure the construct but are part of it” (p. 5). Thus, a network constitutes a system wherein the constituent variables mutually influence each other without hypothesizing the existence of causal latent variables (Schmittmann et al., 2013; Hevey, 2018). From this perspective, questionnaire items refer to the state of a set of personality components that are causally dependent upon one another and form a network. The state of the network is determined by the total activation of these components. High levels of CSA are portrayed when more components of the construct are activated, and the network is pushed toward an anxious state (Borsboom and Cramer, 2013). A network model of CSA would depict the observed variables as nodes connected by edges, which represent statistical relationships between nodes. In this way, the psychological network helps illuminate the morphology of the construct.

A natural corollary of adopting a network approach is the shift in focus of therapeutic interventions. Instead of targeting a latent construct or disorder, interventions can focus upon symptoms and the relations between symptoms (Borsboom and Cramer, 2013). Sport psychologists can direct treatment at the problems faced by athletes, the symptoms themselves, or the causal relations that connect them. Network analysis can reveal *how* these features interact, in contrast to the latent variable perspective, which explicitly prohibits such interactions. In addition, this approach can reveal how the features of CSA might manifest themselves differently in athletes with the same overall scores on state anxiety inventories. To date, researchers have applied network theory to several different psychological constructs (e.g., conscientiousness, Costantini and Perugini, 2016) and disorders (e.g., depression, Bringmann et al., 2015; post-traumatic stress disorder, Ross et al., 2020; trait rumination, Bernstein et al., 2019, and for a review, Fried et al., 2017). This paper is the first to examine the dynamics of the CSA response from a network perspective.

Network analysis also affords researchers the opportunity to examine individual differences in the CSA response. In the competitive state anxiety research, the examination of gender effects has been equivocal. Despite the suggestion that gender does moderate anxiety responses (Martens et al., 1990), subsequent research using the Competitive State Anxiety Inventory-2 (CSAI-2; Martens et al., 1990) has reported no differences (e.g., Perry and Williams, 1998) and others reporting a range of differences between males and females (e.g., Hagan et al., 2017). Research using Cheng et al.’s three-dimensional measure is more limited with only Cheng et al. (2011) examining gender differences and reporting no effect. Consequently, we aimed to explore potential differences between male and female CSA network structures.

One of the challenges facing researchers constructing network models using self-report scales such as the TFAI stems from the design of such scales, which have been constructed to measure underlying dimensions or latent variables (Fonseca-Pedrero et al., 2018; Briganti and Linkowski, 2020). Specifically, the items contained in the scales are often similar and might measure the same construct. Consequently, rather than representing the mutualism inherent in paired connections between nodes within a network, any interaction between items might represent shared variance as the items were designed to measure the same thing (Fried and Cramer, 2017). Researchers have adopted several approaches to overcome this issue. For example, Fonseca-Pedrero et al. (2018), Briganti et al. (2019), and Briganti and Linkowski (2020) chose to estimate a network for the scale items and a separate network for the latent variables the items reflected. Others (Levinson et al., 2018; Bernstein et al., 2019) have addressed this issue of topological overlap in the items using a data-driven approach to reduce the number of items, based upon their similarity, to the extent that they were more confident that the items were not measuring the same symptoms. In this paper, we adopted the latter approach with the TFAI.

The aim of this study is to extend the use of network modeling techniques to the construct of CSA as represented by Jones et al.’s (2019) adaptation of the TFAI in a sample of athletes competing in a range of sports. We first checked that there were no differences between the networks of male and female athletes and then explored the connectivity of CSA as a network composed of its items. We assessed the accuracy of the networks using bootstrapped confidence intervals on the edge weights and used estimates of predictability to interpret the network structures. Finally, we examined the TFAI items to see whether the network items formed distinct communities or sub-networks that corresponded to Cheng et al.’s (2009) three-factor structure or Jones et al.’s (2019) fully differentiated 6-factor first-order structure. We used a community detection algorithm to identify potential communities, which are groups of nodes that are highly interconnected but connected weakly with other nodes or groups of nodes. Importantly, these communities are not formed because of a common cause, instead they “emerge from densely connected sets of nodes that form coherent sub-networks within the overall network” (Christensen et al., 2020, p. 6). If the presence of communities of items was confirmed, we also set out to examine if there were any items that acted as “bridges,” that is processes that are shared by or connect communities. Overall, this examination of CSA is novel and exploratory and is intended to provide a new perspective on the structure of the CSA response.

## Materials and Methods

### Participants

The de-identified archival data came from a research program that previously investigated the competitive state anxiety response (Jones et al., 2019). The sample of 485 British participants comprised 162 male athletes (mean age = 21, *SD* = 4) and 323 female athletes (mean age = 21, *SD* = 3.7) who competed in a range of individual and team sports (males: archery = 24, badminton = 13, basketball = 36, soccer = 39, field hockey = 4, karate = 3, rugby union = 27, volleyball = 15; females: archery = 14, badminton = 7, cheerleading = 5, hockey = 26, karate = 5, netball = 227, rugby union = 30, touch rugby = 9). The competitive level of the participants ranged from club to international. Athletes had an average of 9.79 (*SD* = 5.59) and 9.21 (*SD* = 4.24) years of competitive experience, for males and females, respectively. All participants were English speaking and informed consent was obtained before beginning data collection. Ethical approval for the study was granted by the university ethics committee.

### Measure

The Three Factor Anxiety Inventory (TFAI) modified by Jones et al. (2019) was used in this investigation. The measure comprises 25 items (see Table 1), with 11 items representing the cognitive dimension (worry, 5 items; private self-focus, 3 items; public self-focus, 3 items), 10 items representing physiological anxiety (5 for both somatic tension and autonomic hyperactivity), and 4 items reflecting the regulatory dimension of perceived control. Participants were instructed to complete the measure based on how they felt at that moment, reminded that their data was confidential and that they should answer as openly and honestly as possible. The prospective data were collected approximately 1 h before a competitive performance. A 5-point Likert scale was used (1 = *totally disagree*; 5 = *totally agree*).

**TABLE 1:** Items from the Three-Factor Anxiety Inventory (TFAI).

**Cognitive dimension**
I am worried that I might make mistakes
I am worried about the uncertainty of what might happen
I am worried about the outcome of my performance
I am worried that I might not perform to the best of my ability
I am worried about the consequences of failure
I tend to dwell on shortcomings in my performance
I am aware that I will scrutinize my performance
I am aware that I will be conscious of every movement I make
I am conscious that others will be judging my performance
I am conscious about the way I will look to others
I am worried that I might not meet the expectations of important others
**Physiological dimension**
I feel physically nervous
I find myself trembling
I have a slight tension headache
I feel lethargic
My body feels tense
My heart is racing
My chest feels tight
I feel tense in my stomach
I feel a lump in my throat
My hands are clammy
**Regulatory dimension**
I feel I have the capacity to be able to cope with this performance
I believe in my ability to perform
I am prepared for my upcoming performance
I am confident that I will be able to reach my target

### Item Selection

To deal with the issue of which items from the TFAI to include in the network we used a data driven approach and compared correlations between all items using the *goldbricker* function in R. *Goldbricker* compares dependent overlapping correlations and if the correlations are significantly different then the symptoms being compared capture unique aspects of the CSA response (see Levinson et al., 2018). The data driven approach involved researcher guided judgment to determine (a) the method chosen to compare correlations, (b) the appropriate level of alpha to determine significance, and (c) which proportion of unique correlations was considered necessary to differentiate items (Levinson et al., 2018). The *goldbricker* output is interpreted in a similar way to a scree plot in principal components analysis: decisions are data driven but combined with theoretical judgments regarding the exact cut off points. In the present study, *goldbricker* was set to search for pairs of items that were correlated at *r* > 0.50, with 0.25 as the significant proportion for inclusion and 0.01 as the *p*-value for determining statistical significance (Hittner et al., 2003; Levinson et al., 2018; Bernstein et al., 2019).

### Network Estimation and Visualization

A network consists of nodes and edges. Nodes represent the individual item scores and the edges are connections between nodes. Node placement was achieved using the Fruchterman and Reingold (1991) algorithm, which places more important nodes at the center of the model in terms of connections to other nodes. An undirected weighted network was estimated a Gaussian Graphical Model (GGM) using *qgraph* and regularized using the Least Absolute Shrinkage and Selection Operator (LASSO). The LASSO regularization returns a sparse network structure as it reduces small connections (partial correlation coefficients) between pairs of nodes to zero. The LASSO penalty is typically implemented to overcome the limitation of relatively small datasets used in psychological research to estimate networks (Epskamp et al., 2017). More specifically, we used *qgraph* to implement a graphical LASSO regularization (glasso, Friedman et al., 2008), which is tuned using the hyperparameter gamma (γ) in combination with the Extended Bayesian Information Criterion (EBIC; Chen and Chen, 2008). The hyperparameter controls the trade-off between the inclusion of possible false-positive edges (high specificity, γ values close to 0) and the removal of true edges (high sensitivity, γ values close to 0.5) in the final network (Heeren et al., 2018). We selected a conservative value of γ = 0.5, guiding the EBIC to favor a sparse network structure with few edges. Epskamp’s *bootnet* package automatically estimates this procedure in *qgraph* using the “EBICglasso” default. In the resulting network, edges between nodes signify conditional independence relationships among the nodes, or more specifically, partial correlations between pairs of nodes controlling for the influence of all other nodes (Epskamp et al., 2017). In other words, the relationships between symptoms account for all other relationships in the model, functioning as a large multiple regression. As our data was ordinal, we specified a Spearman’s correlation matrix as the input for network estimation. We also conducted a form of sensitivity analysis to address concerns that specificity in EBICglasso networks can be lower when the network is dense with many small edges, which can lead to false positive identification of the smaller edges (Williams and Rast, 2020). Although our main EBICglasso analysis used a conservative level of the hyperparameter γ, 0.5, to control for potential false positives, we also constructed a more conservative thresholded network that set edge weights to zero when those edge weights were not larger than the set threshold (see Supplementary Materials; Epskamp, 2018). The network structures were visualized using the R-package *qgraph* (Epskamp et al., 2012). Blue lines indicate positive partial correlations and red lines negative partial correlations. More saturated, thicker edges represent stronger relationships. To assess the accuracy of the networks, we first estimated confidence intervals on the edge weights using bootstrapping routines (1,000 iterations) in *bootnet*. Smaller confidence intervals indicate greater accuracy. We then conducted difference tests between all pairs of edge weights.

### Network Comparison

Male and female networks were compared using the Network Comparison Test (NCT; van Borkulo, 2019). Comparison of networks requires groups of equal sizes, otherwise regularization becomes problematic. To overcome the imbalance between males and females in the sample, we reduced the larger female dataset to match the male dataset using random sampling. We then estimated two networks as described for the overall sample. Implemented in R, the NCT, which combines advanced network inference with permutation testing, then evaluated two hypotheses. The first that network strength was invariant across the two sub-networks tested the extent to which the network structures were identical. The second compared invariant global network strength, which examined whether overall sub-network connectivity was equal between the male and female sub-networks. The NCT is a two-tailed permutation test in which the difference between males and females is calculated repeatedly (1,000 times) for randomly regrouped individuals, with the assumption that both groups are equal. The distribution can be used to test the observed difference between the male and female networks, with a 0.05 significance threshold (van Borkulo et al., 2015). As Stockert et al. (2018) noted, the NCT was validated for networks based on Pearson correlations. As we used Spearman correlations to construct our network, we followed the same procedure as Stockert et al. and investigated the similarity between the data’s Pearson and Spearman correlation matrices. The resulting correlation coefficient was *r* = 0.89 and on that basis, we used Pearson correlations to compare the networks of the male and female athletes. The result of the NCT was used to determine whether subsequent network inference would proceed independently for male and female athletes, or whether the sample could be examined as a whole.

### Network Structure and Inference

We estimated node predictability (Haslbeck and Waldorp, 2018) using Haslbeck’s (2020) *mgm* package. Predictability is “the degree to which a given node can be predicted by all the other nodes in a network” (Haslbeck and Fried, 2017, p. 1) and is an absolute measure of interconnectedness as it provides us with the variance of a node that is explained by all its neighbors. It can be interpreted as being analogous to *R*^2^, or the percentage of variance explained. Other measures of network structure and inference are often used in the network literature, for example strength centrality (Boccaletti et al., 2006) and expected influence (Robinaugh et al., 2016), but these only address the relative importance of nodes. As a result, in line with Briganti et al. (2019) we relied upon node predictability to address the issue of node interconnectedness.

### Community Detection

To test whether the 15 items formed a single or multiple communities within the network, we used Exploratory Graph Analysis (EGA; Golino and Christensen, 2020) estimated using the *EGAnet* package within the R environment. *EGAnet* uses the Louvain community detection algorithm, which Christensen et al. (2020) have demonstrated performs comparably or better than the Walktrap or spinglass algorithms that have typically been adopted in the network literature. The structure of detected communities was further explored using standardized node strength and structural consistency was examined using the R package Bootstrap EGA (*bootEGA*; Golino and Christensen, 2020). Standardized node strength can be interpreted in the same way as an exploratory factor analysis load matrix; however, the community loadings are much smaller than the loadings of a traditional factor analysis matrix as they represent partial correlations (Christensen et al., 2020). To interpret these loadings Christensen et al. recommend using effect sizes of 0.10, 0.30, and 0.50, which correspond to small, moderate, and large effects, respectively, however, these recommendations should be used with caution as no norms have yet been established. Structural consistency is the extent to which causally coupled components form a coherent sub-network (community) within a network. To calculate structural consistency, we used the non-parametric *bootEGA* procedure, which computed the proportion of times each community is exactly recovered from the replicate bootstrap samples generated by *bootEGA* (Christensen et al., 2020).

### Bridge Nodes

Using the *bridge* function from the R package *networktools* (Jones, 2020), we used *one-step bridge expected influence*, which is the sum of the edge weights connecting a given node to all nodes in the other community or communities, to identify important nodes that serve as bridges between communities. *Two-step expected influence* extends this measure by taking into account the secondary influence of a node via the influence of those nodes with which it shares an edge. For ease of interpretation, we plotted *z*-scores rather than raw values.

## Results

### Item Selection

The dependent correlation analysis run in *goldbricker* revealed twenty-one pairs of items that were overlapping. One item from each of these pairs was then removed, resulting in the removal of 10 items from the network. The final 15 items are highlighted in Table 2.

**TABLE 2:** Items from the TFAI included in the network analysis following data reduction, including node predictability.

Node label	Item	Node Pred.
**Cognitive dimension**		
MIST	I am worried that I might make mistakes	0.47
UNCER	I am worried about the uncertainty of what might happen	0.39
CONS	I am worried about the consequences of failure	0.38
DWELL	I tend to dwell on shortcomings in my performance	0.30
SCRUT	I am aware that I will scrutinize my performance	0.27
CONS	I am aware that I will be conscious of every movement I make	0.23
OTHER	I am conscious that others will be judging my performance	0.33
**Physiological dimension**		
NERV	I feel physically nervous	0.55
HEAD	I have a slight tension headache	0.28
LETH	I feel lethargic	0.23
TENSE	My body feels tense	0.46
RACE	My heart is racing	0.40
CLAM	My hands are clammy	0.31
**Regulatory dimension**		
CAP	I feel I have the capacity to be able to cope with this performance	0.24
CONF	I am confident that I will be able to reach my target	0.19

*Node Pred., Node Predictability.*

### Graphical LASSO Network

**FIGURE 1:**
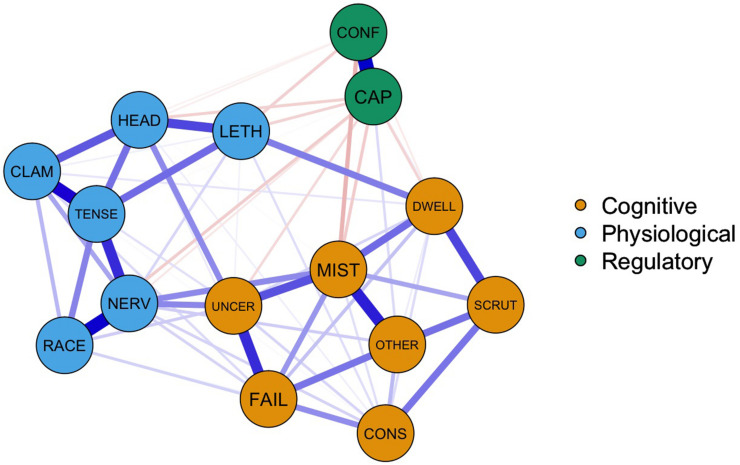
Gaussian graphical model of the final 15 TFAI items. Color groupings correspond to Jones et al.’s (2019) higher order dimensions of cognitive and physiological anxiety and the regulatory dimension. Node labels represent abbreviations for items in Jones et al.’s model (see Table 2).

We produced two networks, a graphical LASSO network, tuned using γ = 0.5 in combination with the EBIC and a thresholded network, which could account for the possibility of detecting a large number of false positives in the EBIC graphical LASSO model. The conservative thresholded method produced a network that produced very few edges that likely misrepresented the true sparsity of the network structure (see Supplementary Material). We used the non-thresholded EBIC graphical LASSO network for subsequent analyses. Figure 1 shows the graphical LASSO network representing the regularized partial correlations among the 15 items of the TFAI. The strongest edges identified were between the 2 nodes representing perceived control (regularized partial correlation: 0.34), between *feeling physically nervous* and my *heart is racing* (0.32), *feeling tense* and *having clammy hands* (0.29), and *worrying about making mistakes* and *being conscious that others would judge performance* (0.26). There were also several negative edges that linked the two perceived control nodes with other nodes across the network. These edges were smaller in magnitude, for example, the largest was between *being confident of reaching one’s target* and *worrying about making mistakes* (−0.08), followed by a series of six relationships where the regularized partial correlation coefficient was −0.05.

### Edge Weight Accuracy

The results of the accuracy analysis (Supplementary Figure S2) indicated that some of the 95% confidence intervals for the edge weights overlapped; however, many of the strongest edges had intervals that did not overlap, suggesting that they were significantly stronger. This interpretation was supported by the bootstrapped edge-weight difference tests (Supplementary Figure S3).

### Network Structure: Gender Differences

The NCT test produced global connectivity values for males and female networks of 5.70 and 5.40, respectively. This difference in connectivity was not significant, *p* = 0.69. Similarly, the test for network structure invariance also failed to reach significance, *M* = 0.24, *p* = 0.32. The networks and edge weight bootstrap results for males and females can be found in the Supplementary Material. The edge weight bootstraps indicated that both the male and female networks were less stable than the main network. As the network structures did not differ for male and female athletes, no further between-gender analyses were conducted.

### Node Predictability

Estimates of node predictability can be found in Table 2. *I feel physically nervous* scored highest on predictability, *R*^2^ = 0.54, indicating that over 50% of variance in this item could be explained by the nodes with which it is connected. Over 40% of the variance in *I am worried I might make a mistake*, *R*^2^ = 0.47; *My body feels tense*, *R*^2^ = 0.46; and *My heart is racing*, *R*^2^ = 0.40, could also be explained by their respective connected nodes. Mean predictability across all of the nodes in the network was *R*^2^ = 0.34 (*SD* = 0.10).

### Community Detection

The EGA detected three communities of nodes that are depicted using the different color schemes in Figure 1. Community 1 contained 3 items relating to worry (*mistakes, uncertainty, consequences*), 3 relating to private self-focus (*shortcomings, scrutinize, conscious*) and the single item representing public self-focus (*others*). Community 2 included the 4 somatic tension items (*nervous, headache, lethargic, tense*) and the 2 autonomic hyperactivity items (*heart racing, hands clammy*), while the final community comprised the 2 perceived control items (*capacity, confident*). Standardized node strength, see Table 3, was used to investigate the contribution of each node to the coherence of each community. Using Christensen et al.’s (2020) guidelines, the loadings for items on each of their respective communities are in the moderate range, with only *lethargic* registering a value of less than 0.20 in its primary community. There are some small cross loadings; *mistakes* with community 3, 0.13; being *worried about uncertainty* with community 2, 0.16; *feeling physically nervous* with community 1, 0.17; and *lethargic* with community 3, −0.11. Most of the cross-loadings are small not only by traditional factor analysis standards but also by partial correlation standards. This is because of the LASSO penalty imposed during the estimation of the network, leaving many nodes unconnected, which results in most of the cross-community connections being small, producing the lower loadings (Christensen et al., 2020). The structural consistency values were high and ranged from 0.81 to 0.88 and 1.00 for community 1, 2, and 3, respectively. Communities 1 and 2 are less consistent that community 3. The small structural inconsistencies in community 1 and 2 are explored in more detail in the Supplementary Materials.

**TABLE 3:** EGA community allocation and standardized node strength for each node.

		Node strength		
	Community	1	2	3
Mistakes	1	0.37	0.06	−0.13
Uncertain	1	0.21	0.16	−0.01
Consequences	1	0.32	0.05	0.00
Shortcomings	1	0.24	0.07	−0.04
Scrutinize	1	0.28	0.00	0.02
Movement	1	0.20	0.06	0.00
Judging	1	0.28	0.02	0.03
Nervous	2	0.17	0.33	−0.01
Headache	2	0.09	0.25	−0.07
Lethargic	2	0.07	0.19	−0.11
Tense	2	0.01	0.45	0.00
Racing	2	0.04	0.25	0.05
Clammy	2	0.02	0.30	0.00
Capacity	3	−0.07	−0.09	0.33
Confident	3	−0.04	−0.03	0.33

### Bridge Expected Influence

**FIGURE 2:**
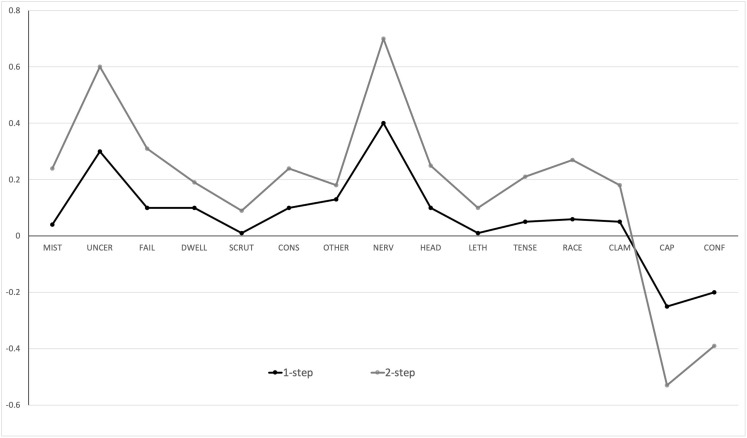
One-step and two-step bridge expected influence.

Estimates of one-step (*bridge* EI1) and two-step (*bridge* EI2) bridge expected influence are plotted in Figure 2. The values reported are standardized expected influence values. Across the 3 communities identified, *I feel physically nervous* from community 2 was the most influential node for both one-step (*bridge* EI1 = 0.40) and two-step (*bridge* EI2 = 0.65) estimates. From community 1, *I am worried about the uncertainty of what might happen* had the highest *bridge* EI1 and EI2 scores; 0.30 and 0.59, respectively. *I feel I have the capacity to be able to cope with this performance* had the highest negative bridge EI1, −0.28, and EI2, −0.55, values. Consistent with expected influence metrics, a Bayesian Pearson’s correlation produced extreme evidence in support of the hypothesis that bridge EI1 and EI2 scores were positively related, *r* = 0.97, BF_+__0_ = 6.75e + 6, 95% CI: [0.88, 0.99], see Supplementary Material for further detail.

## Discussion

To the best of our knowledge, this is the first study to examine the network structure of the competitive state anxiety response. To this end, our study was exploratory in nature. In terms of network estimation, one of the most notable features of the results was the observation that not all of the items were equally important in determining the network structure of CSA, a feature that highlights the value of viewing nodes as processes that can interrelate without reflecting an underlying latent factor (van der Maas et al., 2006). Looking more closely at the relative importance of nodes using node predictability, the high scores recorded for *I feel physically nervous* and *I am worried that I might make mistakes*, indicate that a considerable amount of variation in these symptoms can be explained by connections to other nodes in the network. The interpretation of node predictability must be conducted with the caveat that edges are non-directional (Haslbeck and Waldorp, 2018). In calculating predictability, we assume that all adjacent edges are directed toward that node, but not vice versa. Consequently, Haslbeck and Waldorp note that the predictability of a node acts as an upper boundary for how much it is determined by the nodes connected to it. The two relatively high predictability scores identify symptoms that afford potential opportunities for controllability in the CSA response (Haslbeck and Fried, 2017). If predictability is high, practitioners might control symptoms via adjacent symptoms in the network. For example, feeling physically nervous might be addressed using traditional somatically oriented interventions that target the two symptoms strongly connected to that node: *My heart is racing*, and *My body feels tense*. Feeling physically nervous was also connected to being worried about uncertainty, a cognitive anxiety symptom, so practitioners might also use techniques designed to manage this cognitive symptom in order to help athletes control their physiological anxiety. While other conceptualizations of CSA also feature interactions between cognitive and physiological symptoms, for example, catastrophe models (Hardy, 1996), the interactions described occur at the latent variable level. Network models allow us to see how symptoms interact directly with one another within the overall network structure. The potential to target specific nodes with an intervention, which in turn has a cascading effect to other nodes, might enable researchers to explain how specific interventions prescribed to treat cognitive and physiological anxiety separately according to the matching hypothesis (Morris et al., 1981), can have cross-over effects on different types of symptom. The cross over effects can be more easily explained using network models without recourse to explanations grounded in the shared variance of cognitive and physiological anxiety. In a similar vein, network models also offer a means of highlighting how multimodal treatment packages (Burton, 1990) may help to control cognitive and physiological aspects of anxiety. Feeling physically nervous was also connected to one of the perceived control items, *I feel I have the capacity to be able to cope with this performance*, so strategies to increase athletes’ coping capacity might also prove helpful. One of the lowest predictability scores was for *I feel lethargic*, 0.23. While some intervention via its neighbors might prove marginally fruitful in managing this symptom, one might also search for additional variables outside the network or try to intervene on the node directly. It would, of course, be unwise to make any firm recommendations based on this single study.

Mean predictability across the whole network was 34%, which is a moderate level of predictability compared to values reported in the clinical literature. For example, Fonseca et al. reported that mean predictability in their network of schizotypal traits was 27.8%, while Haslbeck and Fried reported values of 40% for networks of depression and anxiety disorders. High overall predictability can be interpreted as evidence for a network that is self-determined, that is to say, the symptoms are generated by one another. Low predictability is indicative of symptoms that are largely influenced by variables outside the network, for example, biological and environmental variables or additional symptoms (Haslbeck and Fried, 2017). Thus, our results indicate that variables contributing to the CSA response might be missing in the estimated model. Some of this unaccounted for variance might be attributed to the symptoms deleted during the initial item selection procedure, used to ensure that our network contained items that captured unique variance rather than the shared variance inherent in the structure of Jones et al.’s (2019) modified TFAI. The mean predictability score for the network comprised of the original 25-items of the TFAI was 0.42, which indicates that we potentially lost 8% of the network’s overall predictability by reducing the number of items we used in our final 15-item network. We would prefer not to sacrifice the parsimony of the 15-item network for increases in node predictability.

Looking at the overall network structure, the thresholded EBICglasso method produced a very sparse network (see Supplementary Materials). We conducted the thresholded analysis to guard against the possibility that specificity can be lower in dense networks with many small edges, which could lead to a large number of false positive edges (Williams and Rast, 2020). The sparse network produced by the thresholded analysis probably misrepresented the true nature of the network. This is perhaps unsurprising as the thresholded method is much more conservative than the regular EBICglasso, often resulting in low sensitivity, which appears to be the case with the present data. Thus, our choice of the non-thresholded EBICglasso estimation was guided by the very sparse threshold network estimated (Supplementary Figure S1) and by Epskamp (2018), who suggested that for exploratory investigations such as the present study, the original EBICglassso is likely to be preferred, while for higher sample sizes and with a focus on identifying small edges, the conservative threshold method may be preferred.

The absence of any male-female differences in the network supported the only research conducted with the TFAI that has examined this individual difference (Cheng et al., 2011). In a wider context, research conducted with the CSAI-2 over the last 40 years has also failed to find any consistent differences between male and female athletes. A limitation of our analysis in this respect is the relatively small sample size used to compare the male and female networks. As our sample only included 162 male athletes, we reduced the size of the female sub-sample to the same number as the Network Comparison Test is currently limited to comparisons between equivalent groups (van Borkulo, 2019). Further research examining potential differences between male and female athletes that also includes other moderating variables such as skill level and sport type is needed to provide some clarity as to how networks might differ as a function of individual differences.

Community detection analyses revealed three distinct subnetworks. An advantage of our method of community detection, exploratory graph analysis, is the ability of the *bootEGA* function to estimate and evaluate the stability of the identified communities. While previous research has relied upon more traditional walktrap and spinglass algorithms for community detection, these methods are limited to placing items in a single community. For psychological data, where items might be expected to cross load between communities, this might be problematic. *bootEGA* produced structural consistency values of 1.00 for the regulatory community and 0.81 and 0.82 for the cognitive and physiological anxiety communities, respectively. As Christensen et al. (2020) note, there is insufficient research to allow us to make judgments of how high or low the lower levels of structural consistency for cognitive and physiological anxiety are, but we can explore why these communities are more structurally inconsistent. The results of this analysis are presented in the Supplementary Materials. The three communities identified by EGA corresponded to the second-order dimensions of cognitive and physiological anxiety and the regulatory dimension originally proposed by Cheng et al. (2009) and supported by Jones et al. (2019). There was no evidence to suggest that the network could be classified into the six first-order factors that formed part of Jones et al.’s (2019) hierarchical model. Although no previous research has explored state anxiety from a network perspective, Heeren et al. (2018) have examined trait anxiety, noting that the trait response did not decompose into communities or subnetworks and was best represented as a unidimensional construct. Direct comparisons are difficult to make as Heeren et al. focused upon anxiety as a disposition rather than a state and they also chose to measure trait anxiety using the STAI-T (Spielberger et al., 1983), which is a scale designed to measure anxiety as a unidimensional construct. One of the criticisms of the work conducted using network analysis is the use of existing self-report measures and in this respect the estimation of networks can only be as good as the items included in the measure adopted by researchers. Future research might focus on developing a more comprehensive measure by engaging in a rigorous process of identifying self-report, environmental and behavioral factors that can influence competitive state anxiety.

In terms of bridge expected influence, which highlights nodes that have the greatest effect on nodes outside their own community, several symptoms stood out. *Feeling physically nervous* from the physiological anxiety community was the bridge node with largest influence throughout the network, sharing large edge weights with *I am worried about the uncertainty of what might happen*, which was the most influential bridging node in the cognitive community, and *I am worried that I might make mistakes*, also from the cognitive anxiety community. *I feel I have the capacity to be able to cope with this performance* had a bridge expected influence value of −0.53 and Figure 1 illustrates how this node links with other nodes outside of the perceived control community. Although the edge weights are small, the negative associations identify how perceived control might have the potential to exert a dampening effect on both physiological and cognitive anxiety symptoms.

While the present study makes a unique contribution to the large body of literature on CSA and provides a novel insight into the dynamics of the construct, there are several limitations to consider that are in addition to the caveat regarding the interpretation of node predictability and small sub-sample size for the Network Comparison Test, noted above. First, participants were from a community sample of athletes experiencing a range of CSA responses. The network might look different if the study was replicated on a sample of athletes who experience high levels of CSA. Second, it is important not to draw conclusions about the CSA response and its relationship with performance from this data. The data are also cross sectional and collected at one point in time. To more fully examine the anxiety-performance relationship, further work is needed to examine how CSA responds dynamically as a result of increased stress, for example by comparing training and competition responses or by tracking CSA across time to an important event and investigating the impact of any change in CSA on athletic performance. Finally, we do not suggest that the network model presented here definitively captures the CSA construct. The aim of our study was to highlight how network analysis can give us a new perspective on how the component processes of the CSA response cluster and interact, suggesting new approaches to intervention by practitioners.

## Conclusion

In conclusion, this study is the first to provide evidence that competitive state anxiety can be conceptualized as a network system. Our findings add to the growing body of literature that has shown that personality dimensions can be conceptualized in network terms. Further research is needed not only to replicate the present data but also to investigate network dynamics as a function of high and low levels of competitive stress and, crucially, how these dynamics relate to performance. Without the constraint that items reflect one or more latent constructs, we have highlighted some of the implications of adopting a network approach for practitioners; however, much more work is needed before any concrete recommendations can be made. Given the extensive literature on competitive state anxiety, our findings set the scene for novel research directions focused upon model conceptualization and the development of more effective interventions.

## Data Availability Statement

The data analyzed in this study is subject to the following licenses/restrictions: Consent to publish the data was not obtained during the initial data collection. Requests to access these datasets should be directed to RM, rich.mullen@brunel.ac.uk.

## Ethics Statement

The studies involving human participants were reviewed and approved by School of Psychology, University of South Wales. The patients/participants provided their written informed consent to participate in this study.

## Author Contributions

RM and EJ were equally contributed to all aspects of the manuscript. Both authors contributed to the article and approved the submitted version.

## Conflict of Interest

The authors declare that the research was conducted in the absence of any commercial or financial relationships that could be construed as a potential conflict of interest.
