# Supplementary material for: Network Analysis of Competitive State Anxiety
Source: Front Psychol. 2021 Jan 11;11:586976. doi: 10.3389/fpsyg.2020.586976 (PMC7829331; doi:10.3389/fpsyg.2020.586976)
Supplement: Supplementary file 1 [file Table_1.DOCX]

**Supplementary Materials**

This document contains the results of the bootstrapping accuracy and stability analyses for all of the networks from the study. A detailed explanation of how to interpret the results can be found in Epskamp, S., Borsboom, D., and Fried, E. (2017). Estimating psychological networks and their accuracy: A tutorial paper. *Behavior Research Methods*, 1-34. doi: 10.3758/s13428-017-0862-1. Presented here are the thresholded network, and stability and accuracy information for the non-thresholded EBIC glasso network presented in the main paper; including bootstrapped confidence intervals for the edge weights and edge weight difference tests. We also include the networks produced for the NCT test between male and female athletes, follow-up data to support the community detection analysis, bridge expected influence bootstrapped confidence intervals and stability centrality coefficient. The final section contains the robustness check for the Bayesian correlation between bridge EI1 and bridge EI2.

**Figure S1.** *Thresholded graphical LASSO network of the final 15 items*


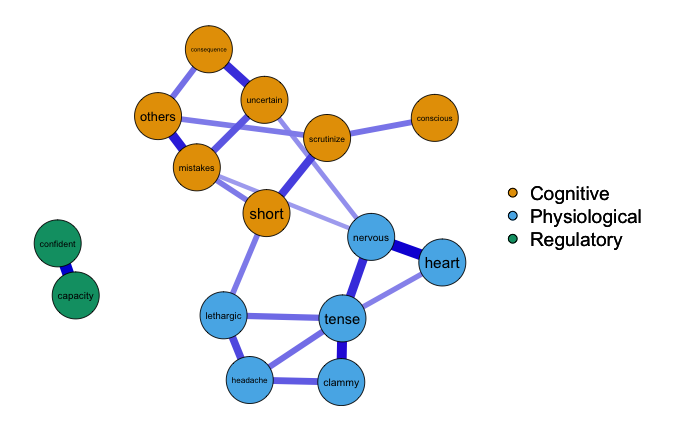


**Figure S2.** *Bootstrapped confidence intervals of estimated edge weights for the total sample for the non-thresholded network presented in the main paper*


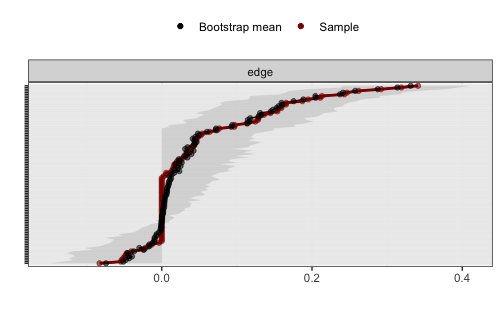


*Note.* The y-axis contains all of the edges in the network with the labels omitted for legibility. The red line represents point estimates of the edge weights, the grey bar their 95% bootstrapped confidence intervals. As Eskamp et al. (2017) note, the CIs are not used to test for significance of an edge being different from zero but are used to show the accuracy of the edge weights. Wide bootstrapped CIs make the interpretation of an edge difficult but do not affect the presence of an edge as model selection as that has already been performed by the LASSO function.

**Figure S3.** *Edge weight difference test*


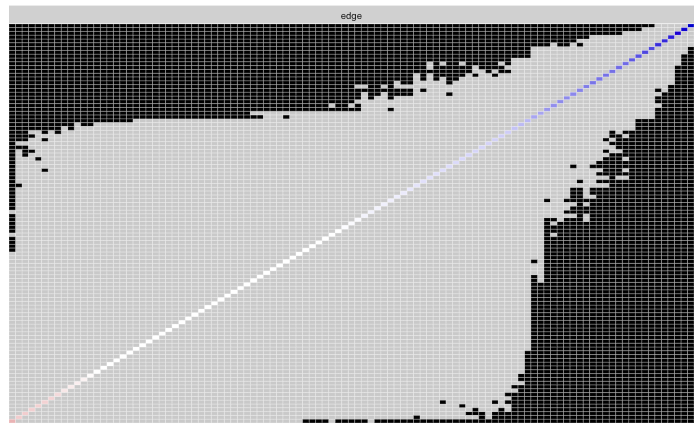


*Note.* Bootstrapped difference tests (α = 0.05) between edges that were estimated at non-zero in the network. Each row indicates an edge. The black boxes represent significant differences and grey boxes represent non-significant differences. The colour in the diagonal corresponds with the edge colours in the original network figures. The test does not control for multiple comparisons and should be interpreted with caution.

**Figure S4.** *Male (L) and female (R) networks*


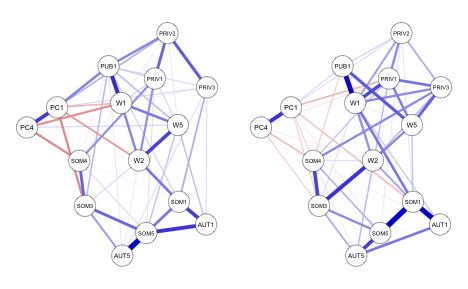


*Note.* Key to abbreviations: W1 = mistakes, W2 = uncertainty, W5 = consequences of failure, PR1 = dwell on shortcomings, PR2 = scrutinize, PR3 = conscious, PU1 = others judging, SOM1 = physically nervous, SOM3 = tension headache, SOM4 = lethargic, SOM5 = tense, AUT1 = heart racing, AUT5 = hands clammy, PC1 = capacity to cope, PC4 = confident.

**Figure S5.** *Bootstrapped confidence intervals of estimated edge-weights for the female (upper) and male (lower) networks.*


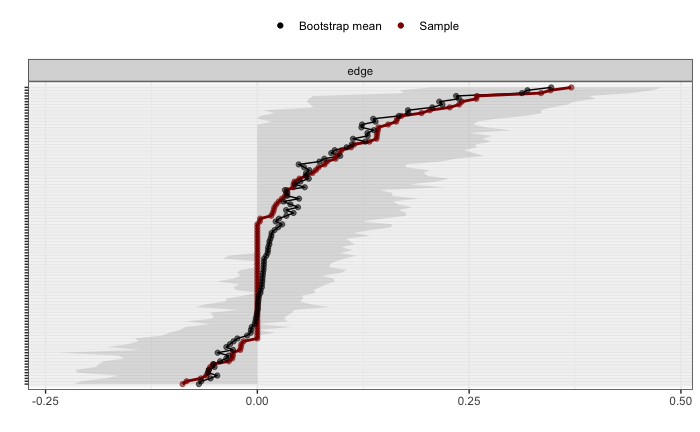


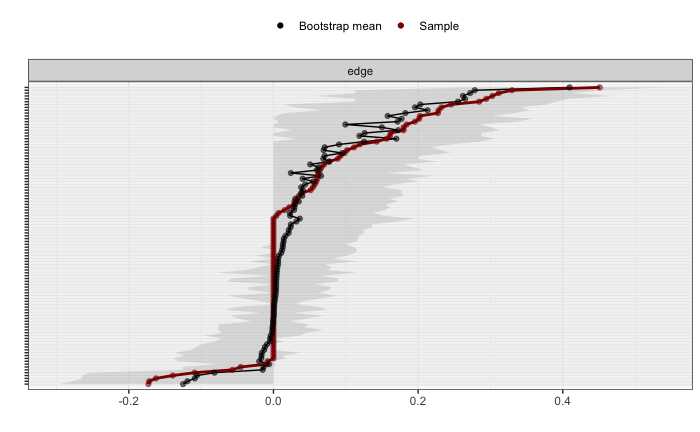


**Community detection**

To further examine the small structural inconsistencies of communities 1 and 2, we used an item stability plot (Christensen et al., in press). The item stability plot (Figure S6) indicates that the majority of the items replicated in their designated community perfectly, with only W2 producing a figure lower than 0.90. Table S?? provides further detail by comparing items’ intra and inter-community replication. The lowest intra-community stability item, W2, loads 14% of the time on physiological anxiety and 4% on a fourth, unidentified community. The fourth community is also responsible for the majority of the remaining inter-community replications, 2% for SOM1 and AUT1 and 8% of the time for SOM3 and SOM4. The presence of a fourth community can be explained by the values in Table S1, which depicts the frequency with which a given number of communities was replicated (Christensen & Golino, 2019). The table confirms the 3-community solution was the most frequent solution produced by the EGA analysis. We see that although the 3-community solution was replicated 80% of the time, 4 communities were replicated 16% of the time, which accounts for the community cross-loadings discussed above. The make-up of that fourth community appears to be primarily driven by community 2, which represents physiological anxiety, with W2 loading on the community with limited frequency.

**Figure S6.** *Item stability plot representing each item’s replication in the original community specified by EGA*


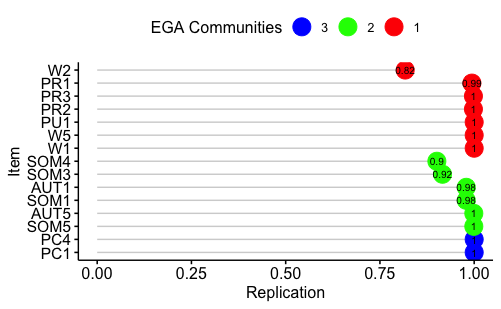


**Table S1.** *Item stability in each community*

|  |  |  |  |  |
| --- | --- | --- | --- | --- |
|  | **Community** | | | |
| **Item** | **1** | **2** | **3** | **4** |
| W1  W2  W5  PR1  PR2  PR3  PU1  SOM1  SOM3  SOM4  SOM5  AUT1  AUT5  PC1  PC4 | 1.00  0.82  1.00  1.00  1.00  1.00  1.00  0.001  0.001  0.007  0.001 | 0.14  0.979  0.916  0.901  1.00  0.979  1.00 | 0.002  0.006  1.00  1.00 | 0.04  0.02  0.081  0.086  0.02 |

**Table S2.** *bootEGA* *dimensions frequency*

| Number  of Factors | Frequency |
| --- | --- |
|  |  |
| 2  3  4  5 | 0.02  0.80  0.16  0.02 |

**Figure S7.** *Bridge expected influence bootstrapped confidence intervals*


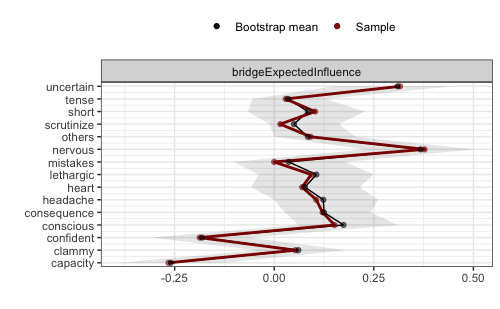


**Figure S8.** *Stability centrality coefficient for one-step bridge expected influence.*


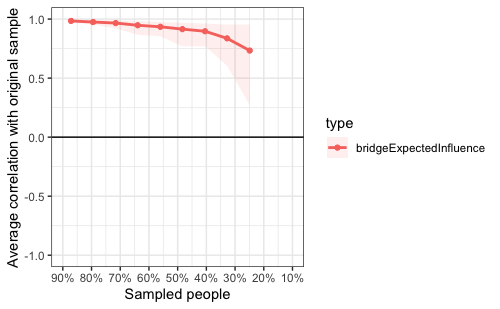


*Note.* The correlation between the original one-step bridge EI, and one-step EI after dropping a percentage of participants at random. The stability centrality coefficient (i.e. % of cases that can be dropped to retain with 95% certainty a correlation of 0.7 of centrality between network estimated on original data and network estimated on subsampled data) for one-step bridge EI was 0.75.

**FigureS9.** *Bayes Factor robustness check for the correlation between bridge EI1 and bridge EI2*


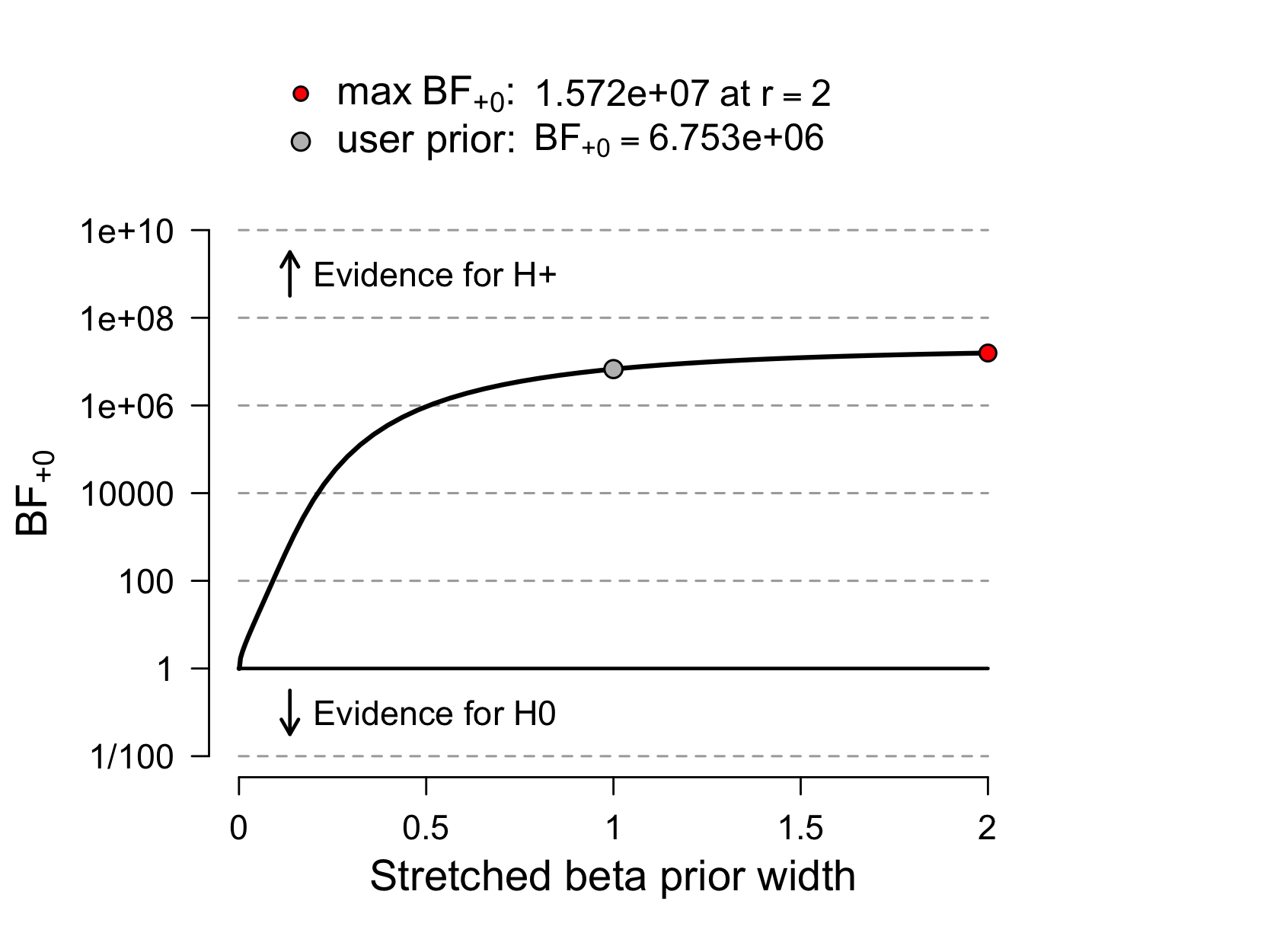


*Note.* We used the default priors in Jeffreys’s Amazing Statistical Programme (JASP Team, 2017) and reported the Bayes factor expressing the probability of the data given H_1_ relative to H_0_, which produced extreme evidence in favour of H_1._ Figure S9 displays the robustness check, which confirms that over a wide range of plausible values for the prior, the data still provide extreme evidence in favour of the hypothesis that bridge EI1 and bridge EI2 are positively correlated.

**References**

Christensen, A. P., Golino, H., and Silvia, P. J. (in press). A psychometric network perspective on the validity and validation of personality trait questionnaires. *Eur. J. Pers*. <https://doi.org/10.31234/osf.io/ktejp>
